# The Ethiopian Health Extension Program and Variation in Health Systems Performance: What Matters?

**DOI:** 10.1371/journal.pone.0156438

**Published:** 2016-05-26

**Authors:** Netsanet Fetene, Erika Linnander, Binyam Fekadu, Hibret Alemu, Halima Omer, Maureen Canavan, Janna Smith, Peter Berman, Elizabeth Bradley

**Affiliations:** 1 Yale School of Public Health and Global Health Institute, New Haven, CT, United States of America; 2 JSI Research and Training Institute, Inc., Boston, MA, United States of America; 3 Harvard School of Public Health, Boston, MA, United States of America; Queensland University of Technology, AUSTRALIA

## Abstract

**Background:**

Primary health care services are fundamental to improving health and health equity, particularly in the context of low and middle-income settings where resources are scarce. During the past decade, Ethiopia undertook an ambitious investment in primary health care known as the Ethiopian Health Extension Program that recorded impressive gains in several health outcomes. Despite this progress, substantial disparities in health outcomes persist across the country. The objective of this study was to understand how variation in the implementation of the primary health care efforts may explain differences in key health outcomes.

**Methods and Findings:**

We conducted a qualitative study of higher-performing and lower-performing woredas using site visits and in-depth interviews undertaken in 7 woredas. We classified woredas as higher-performing or lower-performing based on data on 5 indicators. We conducted a total of 94 open-ended interviews; 12–15 from each woreda. The data were analyzed using the constant comparative method of qualitative data analysis. Substantial contrasts were apparent between higher-performing and lower-performing woredas in use of data for problem solving and performance improvement; collaboration and respectful relationships among health extension workers, community members, and health center staff; and coordination between the woreda health office and higher-level regulatory and financing bodies at the zonal and regional levels. We found similarities in what was reported to motivate or demotivate health extension workers and other staff. Additionally, higher-performing and lower-performing woredas shared concerns about hospitals being isolated from health centers and health posts. Participants from both woredas also highlighted a mismatch between the urban health extension program design and the urban-dwelling communities’ expectations for primary health care.

**Conclusions:**

Data-informed problem solving, respectful and supportive relationships with the community, and strong support from zonal and regional health bureaus contributed to woreda performance, suggesting avenues for achieving higher performance in primary health care.

## Background

Primary health care services are fundamental to improving health and health equity, particularly in the context of low and middle-income settings where resources are scarce [1], Community health workers are widely used in resource-limited settings to promote access to primary health care and improve health outcomes [2]. During the past decade, Ethiopia undertook an ambitious investment in primary health care known as the Ethiopian Health Extension Program [3, 4]. The government in this time has created and deployed more than 38,000 health extension workers, established more than 16,000 health posts and approximately 3,500 health centers, and is now establishing primary hospitals at the district level (referred to as a “woreda” in Ethiopia). Households are organized in to health development army (HDA) for participatory learning and action meetings to actively engage community in health extension program [5]. The vision of the federal Ministry of Health has been to integrate these facilities and functions at the woreda level to ensure access and quality of primary health care serving approximately 100,000 people in each woreda.

Ethiopia has made impressive gains in several health outcomes nationally including maternal, neonatal, infant, and child mortality [6, 7], and the health extension program has been credited with greatly improving utilization of maternal and newborn health services [8–12]. Despite this progress, substantial disparities in health outcomes persist across the country [13]. Previous research in Ethiopia has attributed these disparities to differences in socioeconomic status, health literacy, and availability of primary care across urban and rural settings and across regions, as well as supervision, training, and knowledge of the HEWs [11, 14–22]. Little is known, however, about how woreda-level implementation of primary health care improvement efforts may influence population health outcomes.

Accordingly, we sought to understand how variation in the implementation of the primary health care efforts at the woreda level may explain differences in key woreda-level health outcomes in Ethiopia. Using data from the Health Management Information System (HMIS), we rated woredas as higher-performing and lower-performing on several health measures and then used qualitative methods to examine differences in primary health care structures, practices, and relationships that were apparent between higher-performing and lower-performing woredas. Findings from this study may help policymakers and practitioners more fully understand health systems factors that may contribute to or mitigate health disparities.

## Methods

### Ethics Statement

All research were approved by the Human Subjects Committee at Yale University. The project was found to be of minimal risk and to meet the approval requirements under University IRB policy and 45 CFR 46 as applicable. All participants were provided with an information sheet to inform them about the objective of the research, let them know what data would be collected, how it would be used and disseminated, and any risks that would be encountered by participation. Verbal consent was most appropriate considering no patient-level data was being collected, unique identifiers were assigned to each interview to manage the recordings and transcripts, and all names and other personal identifiers were be stripped from the transcripts. Original recordings and transcripts will were maintained in secure, locked offices and on secure servers by the research team.

Because we obtained verbal consent, documentation of consent was not required. Study procedures were reviewed by the Federal Ministry of Health and relevant Regional Health Bureaus in Ethiopia, and consent procedures were approved by the Human Subjects Committee at Yale University.

### Setting

Located at the horn of Africa, Ethiopia has nine regional states and two city administration councils. The regional states and city administration councils are further divided into woredas of approximately 100,000 people, which are subdivided into kebeles, the smallest administrative unit of government [23], with a population of approximately 5,000 people. Life expectancy at birth in Ethiopia is 62 for men and 65 years for women [24], with 68 per 1,000 live births dying before they are 5 years old. The maternal mortality ratio is estimated at 420 per 100,000 live births; most public health indicators are trending in the positive direction with development. Ethiopia has a three-tiered system of primary, second, and tertiary care [25]. Tertiary care is delivery by specialized hospitals with catchment areas of 3.5–5 million people; secondary care is delivered by general hospitals with catchment areas of 1–1.5 million people, and primary care is delivered by primary hospitals, health centers, and health posts serving 60,000–100,000 or more people at the woreda level. The woreda-level primary health care system [23] typically includes a woreda health office, a primary hospital, at least 5 health centers, 25 health posts, and 50 health extension workers (two per health post).

### Study design and sample

We conducted a qualitative study of higher-performing and lower-performing woredas using site visits and in-depth interviews undertaken in 7 woredas selected to reflect urban and rural woredas, as well as the 4 main regions of Ethiopia and the Addis Ababa city administration **(Table 1)**. We conducted 12–15 open-ended interviews in each woreda for a total of 94 interviews **(Table 2)**. All individual approached for interview agreed to participate after informed consent. Research procedures were reviewed and approved by the Institutional Review Board at the Yale School of Medicine and the Ethiopian Federal Ministry of Health, as well as relevant Regional Health Bureaus.

**Table 1:** Sample woredas by region, performance, and setting.

Region	Performance	Setting	Number of interviews
Oromia	Lower	Urban	13
	Lower	Rural	14
Tigray	Lower	Rural	13
SNNPR	Higher	Urban	14
	Higher	Rural	14
Amhara	Higher	Rural	14
Addis	Higher	Urban	12
Total number of interviews			94

**Table 2:** Participant demographics (N = 94).

	N (%)^1^
**Region**	
Oromia	27 (29%)
Tigray	13 (14%)
SNNPR	28 (30%)
Amhara	14 (15%)
Addis Ababa	12 (13%)
**Woreda setting**	
Rural	55 (59%)
Urban	39 (41%)
**Woreda performance**	
Higher Performing	54 (57%)
Lower Performing	40 (43%)
**Informant position**	
Community member	14 (15%)
HEW	14 (15%)
Health extension program supervisor	7 (7%)
HMIS/referral focal person	14 (15%)
HC director/coordinator	7 (7%)
Woreda health officer	6 (6%)
Zonal administration	6 (6%)
Regional administration	5(5%)
Woreda administrator	7 (7%)
Hospital CEO	7 (7%)
Hospital medical director	7 (7%)
**Informant gender**	
Male	65 (69%)
Female	29 (31%)
**TOTAL**	94 (100%)

To draw the sample of woredas, we classified woredas as higher-performing or lower-performing based on data on 5 indicators obtained from the 2012–2013 Health Management Information System (HMIS) woreda-based planning report; the 5 indicators were: 1) the percentage of pregnant women who have at least 1 antenatal care visit, 2) the percentage of births with a skilled birth attendant, 3) the percentage of infants who receive complete immunization according to World Health Organization recommendations [26], 4) the percentage of woreda households with a latrine, and 5) the percentage of families in the woreda that had been certified as “model families.” The certification as a model family process in Ethiopia [12] is part of national health reforms that encourage families to complete several trainings and demonstrate improved health behavior to earn certification as a model family. Each woreda received a score of 1 through 4 for each indicator, where 1 represented the lowest quartile of performance and 4 represented the highest quartile of performance for their region and urban/rural categorization. The summary performance scores thus ranged from 5 (lowest performance) to 20 (highest performance) relative to their region and urban/rural classification.

We randomly selected woredas from those in the top 5% and in the bottom 5% of performance (based on the 5 indicators) for their region and urban/rural classification to attain a sample of higher-performing and lower-performing woredas. From the sample of higher-performing woredas, we selected the highest performing health center based on data available for the 5 indicators. Likewise, from sample of the lower-performing woredas, we selected the lowest performing health center based on data available for the 5 indicators. In selecting highest- and lowest-performing health posts, data on latrines and model families were missing at the health post level for 7 woredas, 3 of which were selected for the present study. In these cases, we selected health posts based on the 3–4 indicators for which data existed.

With the permission of the regional, zonal, and woreda health offices, we approached clinical and administrative staff in the regional, zonal, and woreda health offices and woreda administration, as well as at hospitals, health centers, and health posts. We also interviewed members of the health development armies (coalitions of community members focused on promoting health) at their work setting or in their community. We selected higher- and lower-performing woredas in urban and rural locations that reflected geographical diversity. In each woreda, we continued to conduct interviews until the point or theoretical saturation [27], or until no new concepts emerged with successive interviews, which occurred after 94 interviews in 7 woredas.

### Data collection

**Fig 1:**
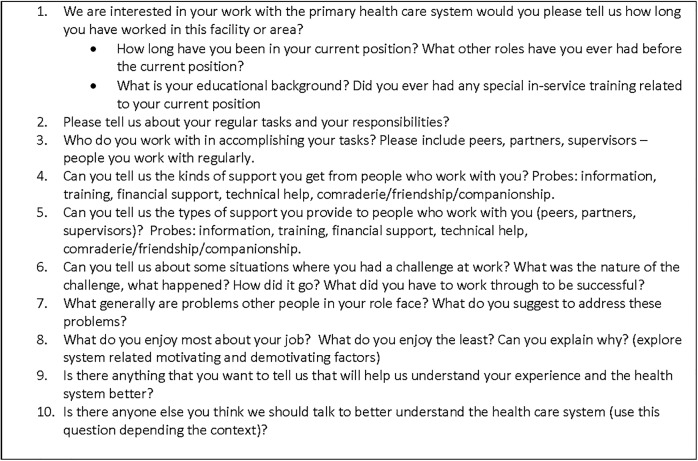
Discussion guide (for clinical and administrative staff).

**Fig 2:**
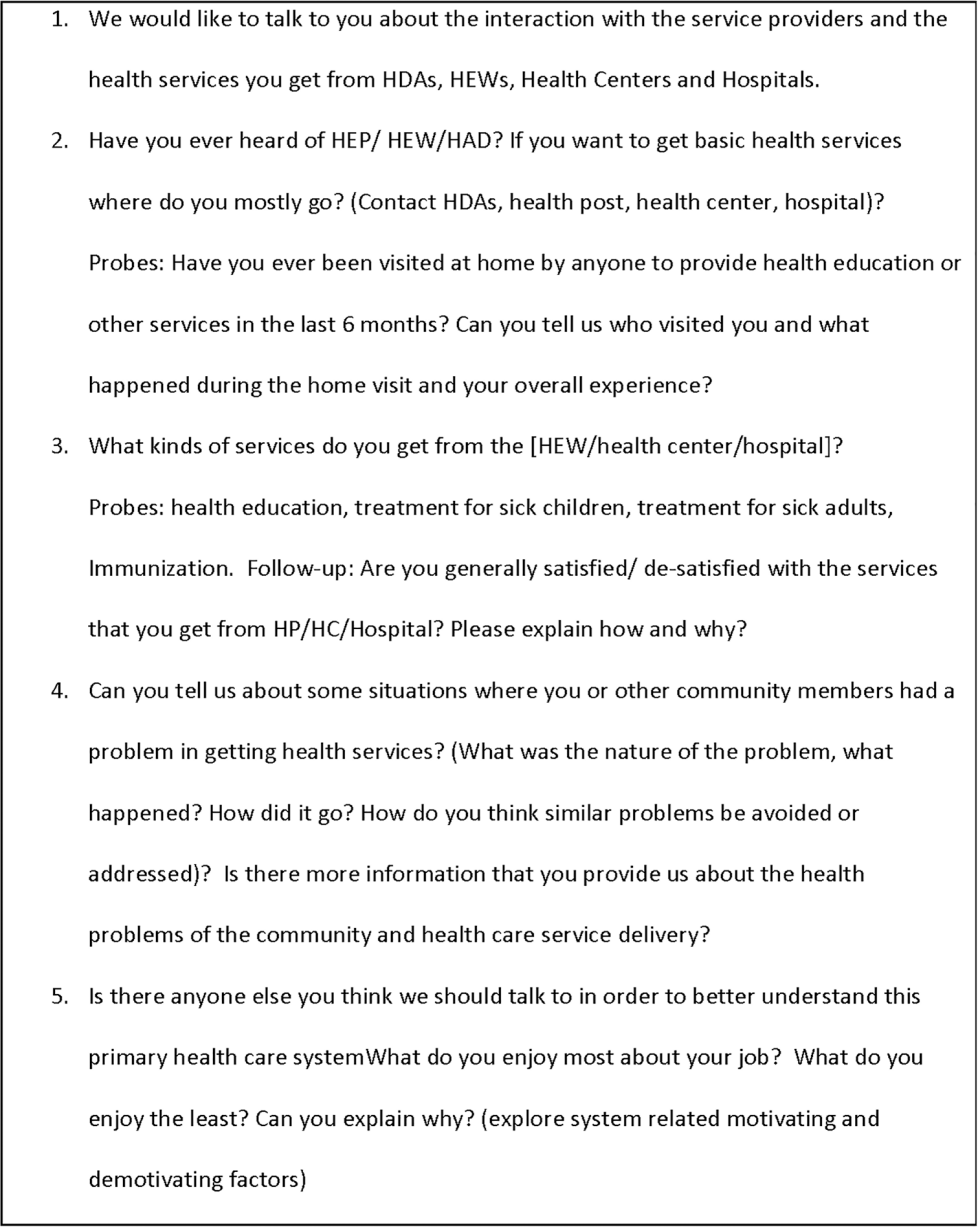
Discussion guide (for community members or service users).

Interviews were conducted face-to-face in the local languages (Amharic, Oromifa, or Tigrina, as appropriate) by research interviewers trained in qualitative interviewing using pretested, open-ended discussion guides **(Figs 1 and 2)**. Interviewers were encouraged to attain vignettes and detailed narratives from participants in order to understand both the overall context and their own experience of their work in the woreda. The discussion guide was designed in to two forms: one applicable for interviews with people in clinical, administrative, and political roles and another applicable for community members.

The interview guide for people in clinical, administrative, and political roles included 5 broad questions: 1) interviewee education and experience, length of service, and major responsibilities, 2) the support the interviewee received or provided while accomplishing their tasks, 3) the challenges encountered and the way they worked through those challenges, 4) the most and least enjoyable aspects of their work, and 5) the interviewee’s perspective on the performance of the woreda. The discussion guide for community members also included 5 broad questions related to: 1) the experience of the interviewee in interacting with health extension workers and other health service providers, 2) the type of health services the community member received from the health extension workers and health service providers in the woreda, 3) the challenges the community member encountered while seeking or receiving health services, 4) other community challenges in maintaining their health, and 5) and the interviewee’s perspective on the performance of primary health care in the woreda.

With permission from each participant, interviews were audio recorded. Recordings were transcribed in the local language and the transcripts were translated into English. Each resulting English-language transcript was reviewed by the interviewer and compared with the audio file for completeness and accuracy of translation and transcription. We also documented participant role, gender, age, and years of experience in their current position.

### Data analysis

The data were analyzed using the constant comparative method of qualitative data analysis [28–30] in which successive transcript are read line-by-line and coded for recurrent concepts using an iterative process. We used an inductive, or grounded, approach [29], building the codes from the data. Three independent researchers reviewed the first several transcripts line-by-line identifying concepts to begin the code sheet. The code sheet was augmented with analysis of successive transcripts, and early transcripts were re-coded to ensure comprehensiveness. The three coders reconciled discrepancies through a series of meetings and communications using negotiated consensus. With the final code sheet, researchers applied appropriate codes to chunks of data (i.e., sentences or paragraphs of transcribed text) and integrated all data coded with like codes. (See code sheet in supplemental material). Reading across codes, we then integrated the codes into broader, recurrent themes with careful attention to disconfirming evidence, as recommended by experts in qualitative data analysis [25, 27–31]. In addition, we examined patterns of concepts and themes as they varied between the higher-performing and lower-performing woredas as well as across urban and rural woredas. We used Atlas.ti version 7.1.7 software to facilitate coding and retrieval of data for analysis.

Several techniques were used to ensure that data analysis was systematic and verifiable, according to state-of-the-art qualitative methods [27, 28, 30–33]. These included 1) consistent use of a discussion guide, 2) interviewing multiple respondents at each site for triangulation, 3) audiotaping and independent preparation of the transcripts, 4) coding and analysis of the data using an explicit coding structure developed and applied consistently by research team, 5) multiple coders with varying relevant backgrounds and perspectives, 6) explicit consideration and discussion of discrepant interpretations resolved by negotiated consensus, and 7) the creation of an analysis audit trail to document analytical decisions.

## Results

### Description of the sample

Participants were drawn from higher-performing and lower-performing woredas representing urban and rural woredas from across 4 regions and Addis Ababa **(Table 1** and **Table 2).** The mean summary performance score did not vary significantly between urban and rural woredas (P-value = 0.87); however, compared with rural woredas, urban woredas had significantly higher rates of skilled birth attendance (50% versus 21%, P-value = 0.01) and higher proportions of households with latrines (81% versus 70%, with borderline significance P-value = 0.05). Antenatal care rates, immunization rates, and proportion of households that were certified as model families were all higher in rural compared with urban woredas, but the differences were not statistically significant (data available from authors).

Differences in performance on the 5 indicators and the mean summary performance score for the highest performing 5% and lowest performing 5% of woredas are shown in **Table 3.** Representatives from both higher- and lower-performing sites described positive changes associated with the health extension program. We noted several key distinctions between higher-performing and lower-performing woreda primary health care systems, as well as recurrent themes observed in both higher- and lower-performing woredas **(Table 4A and 4B)**.

**Table 3:** Performance on five key indicators in highest performing 5% and lowest performing 5% woredas in Ethiopia (N = 37 woredas in each group).

Indicator	Higher Performing (n = 37)Mean (Standard deviation)	Lower Performing (n = 37)Mean (Standard deviation)	P-values(T-test)
Antenatal care (1 visit)	99% (2%)	66% (23%)	<0.001
Skilled birth attendance	42% (27%)	13% (17%)	<0.001
Infant complete immunization	96% (7%)	58% (17%)	<0.001
Households with latrines	93% (10%)	52% (4%)	<0.001
Percent model families	83% (20%)	33% (24%)	<0.001
Mean summary score	19.0 (1.0)	6.8 (1.3)	<0.001

Source: HMIS data; September 2012—August 2013

**Table 4:** Comparing higher-performing and lower-performing primary health care units.

**A. Contrasts between higher-performing and lower-performing primary health care units**		
**Theme**	**Higher-performing primary health care unit**	**Lower-performing primary health care unit**
Use of data for problem solving and performance improvement	Routine use of data in problem solving and performance improvement; learning from the success of others; regular and structured data validation and feedback loops	Lack of data for consistent reporting, evaluation, and problem solving; mistrust of data quality
Relationships between health centers, HEWs, and the community	Respectful, supportive, frequent contact between HEWs, community, health development army, and health center staff	Strained, distant, little support in the community, from the health development army, or from the health center staff
Coordination and support from higher-level regulatory and financing bodies	Collaborative, sharing information, supportive with budget and training	Limited communication and coordination, stressful relationships, low contact
**B. Similarities across higher-performing and lower-performing primary health care units**		
Motivation	Staff motivated by success in helping individuals and community; staff demotivated by perceived lack of career growth, inadequate financial compensation, limited role clarity, and inattentive or non-supportive supervision	
Hospital engagement	Lack of communication from hospital to promote follow-up after discharge; lack of communication from the health centers when planning for referral to hospital; community accessing hospitals directly without first accessing the primary health care system.	
Urban communities	More wealthy urban community members’ preference for access to physicians for primary health care; Urban community members work and spend much time outside of the home; Some feelings of lack of respect from urban-dwelling clients toward health extension workers, based on education or socioeconomic status.	

#### Descriptions of positive changes

Participants from both higher- and lower-performing sites described positive changes in their communities associated with the health extension program. This finding was apparent across rural and urban settings, and distance from major towns did not seem to influence the experience. Participants gave detailed and meaningful examples, especially related to hygiene and environmental sanitation. As community members from both lower and higher performing sites described:

*Today there is nobody who don’t have latrine*, *[we do] it by educating and by inspiring*. *We have learnt to teach*, *in order to make nobody defecate in the field”* (Health development army member; higher-performing, rural; P33, W2)

*We are changing ourselves*, *our children and our environment with the education they are giving us*. *In the past*, *only those who had some education and awareness were digging garbage pits and latrines*. *Nowadays*, *following the education they [HEWs] provide at the lower levels*, *we dig garbage pits and latrines separately*. *They come to our neighborhood and teach us where to burn dry garbage and where to dump the wet one…I can say that I have gained strong awareness from them*. (Health development army member; lower-performing, urban; P30, W4)

In addition to experiencing improvements, people interviewed in both higher-performing and lower-performing primary health care units described impressive cross-sectoral investments and collaborations that were undertaken in a collaborative, complementary way. Several participants reflected that agriculture and education were viewed as contributing to the health goals of the community, as described by a woreda health office head in a higher-performing woreda.

*The education sector also has responsibilities like educating children about pregnancy follow up*, *immunization and so on and so forth through education awareness will be created in schools*. *Moreover*, *in the agriculture sector it will enable the community to eat a balanced diet; this is one package of the health sector…there is [also] what we call ‘protein corn*.*’ This means it is a corn that is rich in protein*. *Therefore*, *if the farmer produces and uses this protein corn*, *then the farmer has gotten protein*. (Woreda health office head; higher-performing, rural; P9, W2)

People interviewed highlighted that health would be achieved most effectively through contributions from different sectors in a holistic approach instead of the health sector working independently. Collaborating with non-health sectors was viewed as central to the woreda health office’s work.

*We do not say this concerns me*, *you*, *or this belongs to health or to agriculture and so on*. *Since the kebele’s activities are evaluated as a structured-whole*, *focusing on this activity or that [activity] alone is not enough to achieve our goals*. (Health center director; lower-performing, rural; P3, W5)

#### Contrasts between higher- and lower-performing woredas

Despite some common experiences, substantial contrasts were apparent between higher-performing and lower-performing woredas, and these differences were pertinent in both urban and rural settings. Prominent among these contrasts were the use of data for problem solving and performance improvement; collaboration and respectful relationships among health extension workers, community members, and health center staff; and coordination between the woreda health office, and zonal and regional health bureaus.

**Use of data for problem solving and performance improvement**: People interviewed from higher-performing primary health care units reported the active use of data for problem solving, and the use of root cause analyses as a foundation for continuous improvement efforts. Feedback, planning, and supportive supervision emerged as important aspects of project work, as described by a health extension worker supervisor in a higher-performing rural woredas:

*We bring the [evaluation] findings to the health center after the supervision*. *We sit with the head of the health center and discuss the [ones who have achieved the] best performances and identify gaps*. *Our main principle is to make the best performances continue*. *In addition*, *we discuss* *why* *the gaps were created and how we are going to correct them*. *We develop feedback on how the work should proceed; then we try to reach a consensus with the health extension workers*. (Health extension worker supervisor; higher-performing, rural; P6, W2)

In some examples, problem solving in higher-performing woredas involved learning from other regions, as described by a health center director from a higher-performing urban woreda:

*In the past*, *mothers used to be sent home if they were diagnosed to have false labor and the real thing happens while they were at home and they deliver there*. *To tackle such problem we have adopted what the Tigray region is already doing that is we have waiting area for those diagnosed false labor*. *In this waiting area there is coffee ceremony*, *porridge and oatmeal will be prepared there while she is being followed*. *This has created a sense of home environment and a feeling of being among family*. *The mothers stay till they give birth here*. (Health center director; higher-performing, urban; P24, W7)

Moreover, people interviewed in higher-performing woredas remarked on routine procedures used to improve workers’ performance. These included data monitoring and feedback, recognizing good performance, and teaching others about what they viewed as best practices or ideas worth scaling up. A woreda administrator from higher-performing rural woreda said:

*There is a command post evaluation meeting with the zonal leaders once or twice a week*. *There is a feedback session during the planning phase [to see] if the planning is exaggerated or under planned*. *During mid-term evaluations*, *[zonal leaders] attend the sessions and give us their constructive comments*. *In addition*, *they do the field assessments*. *During the field assessment visits*, *they crosscheck our reports with the actual activity on the ground*. *Basing the comments from the zone*, *if there are gaps*, *then we will fill those gaps together*. *And if there are best practices*, *we will try to illuminate them more*. (Woreda administrator; higher-performing, rural; P8, W2)

Supervisors across administrative levels in higher-performing woredas reported using checklists to monitor employee trainings and performance. The process for supervision was described as follows in one higher-performing rural woreda:

*For each kebele [health post]*, *professionals are assigned from the health center*. *There are specific days that they provide supports*. *This is listed; based on this [schedule] they go and provide the needed support and come back*. *There is a checklist prepared by the health center and they will support based on the list of the activities on the checklist*. *The professionals will stay all day providing support*, *assessing what has been done*, *solving the challenges faced*, *and making discussion on the findings*. *In addition*, *we [the health center] evaluate the overall work of HEW every month*. (Health extension worker supervisor; higher-performing, rural; P6, W2)

In contrast, lower-performing woredas struggled to have high quality data reports. Participants described that they lacked the resources needed to collect timely and accurate data (e.g., limited information technology, limited educational background on data collection and reporting). Additionally, participants underscored the irregularities of reporting, suggesting that even if data were collected accurately, it was not always reported consistently.

*What disappoints me firstly is a false report*. *There is a wrong evaluation given sometimes and false statement is presented without being filtered*. *There are instances whereby the institution and individuals are wrongly awarded*. *Some even add up false figures to state the prevention of epidemic or other work but as a person who has been on the task for many years I know when false figures are reported*. (Zonal health department head; lower-performing, rural; P85, W5)

Furthermore, when reports and data were available, the data were not always used for learning, problem solving, and performance improvement. The focus was on being sure reporting requirements to the woreda were met without understanding that such data could be a powerful tool for inclusive performance improvement efforts at the woreda-level primary health care system overall. Woreda officials were described as requested data directly from health extension workers and bypassing the HMIS focal person at health center, resulting in missed opportunities for evidence-based problem solving and collaboration:

*Sometimes conflicts arise when you strive to maintain regulations and procedures*. *For instance there is a report we file every 15 days or every month and the woredas cut you out and take the report from health extension [workers] when you ask them to uphold the procedure*. *The health extension [worker] is afraid of the woreda officials and therefore they give them their reports*. *Their report is therefore not available at the health centers but it is there with the woreda and the zone; the zone later comes back to you for evaluation and asks you to present that report which we don’t have*. (Health extension worker supervisor; lower-performing, urban; P36, W4)

**Relationships between health centers, health extension workers, and community**: Staff and community members in higher-performing PHCUs described effective collaboration among the health extension workers, kebele councils, health centers, and health development armies. In higher-performing woredas, health extension workers described being supported by the community with needed materials and resources:

*If there is a job that needs to be done in the community we first discuss and reach on consensus with the community members*. *We will say that we plan to do this and this activities*, *and we want you to help us in terms of manpower*, *resource and money*. *Then they help us*. (Health extension worker; higher-performing, rural; P6, W2)

Furthermore, health extension workers in higher-performing woredas reported feeling respected and listened to by the community, as suggested in the following statement from a health extension worker in a higher-performing woreda:

*The people in my site…usually welcome me into their home and offer me cup of tea or coffee*. *It is only sometimes that I face people who resist my service and my persistence win them over through continuous visiting*, *involving other members of the community to convince them and giving them brochures and fliers for them to read…I’m continuously following them up*. (Health extension worker; higher-performing, urban; P45, W7)

In contrast, people in lower-performing woredas reported conflict arising in their everyday work, reflecting strained relationships with the community and with other health professionals. Commenting on the poor relationships with the community, one health extension worker from a lower-performing, rural woreda described:

*When we get to their homes and tell them to maintain sanitation*, *there are some people accusing us of trespassing and entering a private property without court order; they may also attempt to beat you up*. *What you must do here is that you must be polite enough to talk to them and convince them as this challenge may come again tomorrow*. (Health extension worker; lower-performing, rural; P36, W4)

Woreda officials and health extension workers in lower-performing woredas also reflected on the prominence of traditional practices and cultural norms that challenged the relationships between the community and the health extension workers.

*There is a lack of understanding with the society in terms of prevention as the awareness was dominated by the traditional practices that prevailed for centuries with the society*. *For instance they don’t take pregnant women to heath facilities for follow-up and delivery as they insist to give birth at home*. *Also concerning toilets and sanitation*, *there is a trend of not quickly getting used to a new approach by quitting the old tradition*. (Woreda administrator; lower-performing, rural; P82, W6)

**Coordination and support from the higher-level regulatory and financing bodies**: In higher-performing woredas, the woreda health office generally described the relationship and support from the zonal or regional health offices as effective. Support included transparent communications about budget decisions, new standards or protocols, and performance expectations. Supervision was seen as regular and supportive with collaboration on improvement efforts, such as reducing vacancies, enhancing transportation resources, or ongoing training of staff. As one zonal-level health officer described the support from the region to an urban woreda,

*We communicate our needs and the regional health bureau leads these [efforts]*, *training health professionals and health extension workers and also organizing the health facilities*. *It provides manuals and guidelines and conducts capacity building works by allocating full budget for training*. *It also provides follow up and…the review is all inclusive*. *The regional health bureau facilitates health extension festivals…* (Health officer; higher-performing, urban; P71, W1)

Such support was apparent in the descriptions of the support from the regional and zonal levels for higher-performing rural woredas as well. In the rural woredas, support for planning and evaluation of performance as well as filling gaps in capacity of primary health care personnel were highlighted as critical inputs. One health officer who worked to link the zone and the woreda described the support from the region,

*The region makes all types of support*. *The first is technical support starting from planning*. *The other one is closing part in which every activity of health care services are evaluated in the presence of different professionals or management bodies from the district and the zone*. *These supports cover all segments of our activities to fill skill gaps*, *provide trainings*, *and provide input supports*. *[The regional health bureau people] go to health post and household level and evaluate what is done*. *This is a very impressive act of inspection and evaluation*. *Lastly they discuss the findings…and [provide] malaria prevention support*, *diagnosis kits*, *medical furniture*, *and diagnosis utensils*. (Health officer; higher-performing, rural; P78, W3)

In contrast, relationships between the woreda-level and higher zonal and regional levels were described as distant, with little contact between focal persons working at each of the administrative levels. One zonal coordinator from lower-performing rural woreda expressed the stressfulness of poor relationships.

*Sometimes when you do good work but you are told otherwise; it disrupts the peaceful work conditions…It [is] a disappointment and discomfort when your superior is simply angry with you at your work without understanding the reasons…a work leader who gives morale to work together is really encouraging for me*. *Personally I think it is better to be invited and encouraged to work together instead of being given an order to do some job*. (Zonal health extension program coordinator; lower-performing, rural; P62, W5)

Similarly, a Health Management Information System (HMIS) focal person from lower-performing urban woreda described feelings of the woreda and kebele levels being abandoned or forgotten by higher levels:

*Whenever directions are sent from [the regional health] bureau*, *they just leave it at zone level; they do not take it down*. *For example*, *we heard when we went to ask our salaries that there are some directions sent recently*. *Let me tell you by relating them*. *When we ask them*, *they tell us that it has already come*. *They say*, *“Here it is*,*” but they do not give it to you*. *They tell you*, *“Here it is*,*” but it has no elaboration*. *They don’t give you the information properly and clearly*. *There the guidelines are*, *the directions that concern us*, *but they conceal them from us*. (HMIS focal person; lower performing, urban; P20, W4)

#### Similarities across higher-performing and lower-performing woredas

We found similarities in what was reported to motivate or demotivate health extension workers and other staff, although motivating factors were more prominent in the higher-performing woredas. Additionally, higher-performing and lower-performing woredas shared concerns about hospitals being isolated from health centers and health posts. Participants in both higher-performing and lower-performing woredas also highlighted a mismatch between the health extension worker preparation and the urban-dwelling communities’ expectations for primary health care.

**Motivation of health workers**: In both higher-performing and lower-performing primary health care units, healthcare workers were motivated by the pride and pleasure of helping patients and serving community members. People recalled their happiest and most fulfilling moments as when they saw individuals recover from illness or whole communities change their health behavior or outcomes, as one health extension worker from a lower-performing rural woreda reflected:

*I am happy for contributing toward reducing mothers and children death; we haven’t had mothers dying here with us*, *but we used to lose children*. *After 2005 we have seen special changes*, *and home delivery rate is getting to zero*. *Delivering a child at home is considered almost a taboo these days…We used call on households for vaccination*, *but today there is no need to call for vaccination as [people] come themselves on the 27*^*th*^ *day*. *This is what makes me happy*, *a change of attitude*. (Health extension worker; lower performing, rural; P83, W6)

Individuals in higher-performing and lower-performing woredas also shared a common view of what demotivated them. Participants described perceptions of limited career growth opportunities and inadequate financial compensation. In addition, staff reflected on the demotivation caused by poor supervision, including both inattentive supervisors and lack of clear role definition by supervisors. From a lower-performing urban woreda, one health extension worker said:

*There is no one who follows up on our performance and gives us moral support and criticizes our weaknesses…We cannot be successful without follow-up and encouragement*. *You begin with courage and determination*, *but due to the lack of moral support you become discouraged*. (Health extension worker; lower performing, urban; P28, W4)

From a higher-performing rural woreda, one health center staff member voiced frustration in not having clarity about his job responsibilities:

*[HMIS focal persons] are expected to do the secretarial work*, *the archives work in addition to their assignment*. *Their major problem is that they don’t know what is expected from them*. *There has to be a job description*. *They are asking their bosses to tell them what to do and what is expected from them*. *One of them will come and say do this and the other will say another…the major problem in many places is lack of job description*. (Health center staff; higher performing, rural; P7, W2)

**Weak connections with hospitals and other components of primary health care**: Participants from all levels and across both higher-performing and lower-performing primary health care units noted gaps in the linkages between hospitals and health centers or health posts. Health center staff, health extension workers, and community members reported vignettes of patients being discharged from the hospital back to the community without sharing information about needed follow up, as illustrated by this quotation from a health center director working in higher-performing rural primary health care unit:

*There is no feedback from [the] hospital that says you have sent such patients and [we] have reached this result…We don’t know whether the patients are dead or alive*. *For example*, *there are poisoned patients*, *which were beyond our capacity*, *and we referred them*. *We hear from others that some of them died*. *Otherwise they don’t inform us through feedback*. (Health center director; higher performing, rural; P32, W2)

Conversely, hospital-based staff complained that patients were often referred to the hospital without prior notification and at times when no beds were available. Some indicated that patients unnecessarily bypassed health centers and accessed the hospital directly despite their condition being treatable at the health center as described by a referral focal person from lower-performing rural woreda.

*Referral linkages are mostly very poor…In relation to delivery*, *they send cases to which solution can be sought at the health centers*, *[and there are] shortcomings in the information about the patient that they are supposed to record and send to us*. (Hospital referral focal person; lower performing, rural; P15, W5)

Although a minority perspective, one hospital medical director in an urban setting described effective linkages, including training and financial support from the hospital to the health center; these linking processes were implemented by an exemplar hospital, as described by the medical director in a higher-performing urban woreda:

*Our hospital is a “LEAD” hospital*, *and we have monthly meeting with health center heads and delivery room workers*. *We discuss major challenges and how to tackle the challenges*. *They present what they have been doing in the past one month like how many they referred*, *how delivery service [was] conducted*. *We have a liaison officer who compiles all referrals from the health centers*, *and summary reports are given to the health centers*. *The mid-wives at health centers come here and get trained on …basic essential obstetric care and newborn care*, *and we have strong relationships*. *We give them supplies*…*We know what support they require to deliver better service*. *And we will do all possible to make that a reality*. (Hospital medical director; higher performing, urban; P47, W7)

**Health extension workers’ challenges in urban communities**: Health extension workers and others interviewed suggested a mismatch between what the health extension workers are prepared to offer and what the urban-dwelling, higher-income community wants related to health care. Urban dwellers were described as wanting access to clinical care with a physician, not a health extension worker, even in cases when the health extension worker was a nurse. As stated by people interviewed in higher-performing urban woreda:

*The [higher-income] group will create a challenge*. *They may claim to have their own doctor*, *and they don’t want to spend their time with the health extension workers*. *They may not respond when their door is knocked*. (Woreda administrator; higher performing, urban; P50, W7)

*Most [urban] people think that they know a lot*, *and when you tell them about the washing facility after [using the] toilet*, *most of them think that it is a rural thing to do…there are cases when people disappear*, *hide*, *or abandon their place whenever you attempt to approach them*. (Health extension worker; higher performing, urban; P69, W1)

Another challenge in the urban settings was described as the diversity of people and their activities, and difficulty finding them at home, as illustrated by this quotation of a supervisor of health extension workers in a higher-performing urban setting.

*The complex socio-cultural diversity and economy [is a challenge]*. *If you go to rural areas homogeneity is more observed in the form of culture and language*, *and it is relatively easier there*. *For example*, *there could be a religious holiday and you could get all villagers assembled*. *But here they [urban residents] work 24 hours and asking health extension workers to find them during weekends is very difficult*. (Health extension worker supervisor; higher performing, urban; P49, W7)

Last, participants reported that more educated urban community members seemed to look down on health extension workers and not value their work, as described by a health extension worker from a lower-performing urban setting:

*They consider themselves educated and they speak to you rudely when you attempt to advise them*. *They consider us nagging*. *They avoid us when we try to approach them*. (Health extension worker; lower performing, urban; P39, W4)

## Discussion

The success of Ethiopia’s health extension program has been well documented [8–12], although health disparities persist [13] with variable performance across the country. In a close examination of structures, practices, relationships among the different components of the woreda-level primary health care system, we found 3 prominent distinctions between higher-performing and lower-performing woredas. Higher-performing woredas had greater use of data-informed problem solving, more respectful and supportive relationships with the community, and stronger support from zonal and regional health bureaus in terms of perceived transparent communication, financial support, and technical inputs. Although much of the previous literature on primary health care improvement has focused on technical inputs as paramount to building primary care systems, our work suggests that more fundamental management and governance capacity is paramount to achieving top performance. Our findings extend a growing body of work [34–37] that identifies the importance of data-informed management and governance capacity in improving primary health care systems in low-income settings.

As important as the contrasts between the higher-performing and lower-performing woredas are the similarities. These highlight the challenges that persisted across diverse settings, despite the extensive investment in Ethiopian primary health care resources over the last decade. One of these challenges involved motivation of staff, and the findings suggest that staff are quite uniform in what motivates or demotivates them. Staff motivation to help their community and see improvements in birth outcomes, vaccination rates, and overall community health was high, but the scarcity of financial compensation and career progression continued to reduce motivation. The finding is consistent with former work concerning community health workers [38–41] and underscores the importance of recent efforts to implement career ladders and enhanced training levels within the Ethiopian Health Extension Program. Consistent with other studies highlighting poor supervision as a key barrier to community health workers’ performance [41–43], our data suggest that improved compensation and supervision (e.g., role definition, clarity in expectations, and recognition for good performance) might increase health extension worker motivation. A second challenge, critical for all health systems, is how best to integrate the hospital while retaining focus on preventive, rather than curative care.

Differences in the recurrent themes between urban and rural woredas based on the qualitative data were not prominent, although quantitative data indicated that urban compared with rural woredas have higher rates of skilled birth attendance and higher proportions of households with latrines. Urban and rural woredas reported many similar experiences; however, unlike participants from rural woredas, participants from urban woredas noted more limited health extension worker access during the day to community members (who were often working or out of the home), and greater challenges related to urban dwellers preferring more sophisticated health care services than available from the health extension worker.

As low-income countries undergo economic development and expand access to hospital care, a balanced investment strategy to address both social and medical determinants of health is critical but difficult to sustain. Last, increased urbanization has created opportunities and challenges for the Ethiopian health extension program as would be expected. We did not find prominent differences in recurrent themes between the rural and urban settings, except in the case of more limited health extension worker access during the day to community members (who were often working or out of the home), and challenges of urban dwellers preferring more sophisticated health care services than available from the health extension worker. Our findings suggest that enabling urban health extension workers to accommodate the work hours of employed populations and equipping the workers with greater clinical capacities and may increase their impact. Creative leadership at all levels is needed to maintain and improve primary health care in the context of the accelerated development in Ethiopia.

Our findings should be interpreted in light of some limitations. First, this was a mixed methods study relying on qualitative methods and hence we cannot establish statistical associations. The findings therefore should be understood as exploratory and hypothesis-generating; nevertheless, we applied several methods to ensure the rigor and reproducibility of our findings [27, 28, 30, 31] including the consistent use of a discussion guide applied in local languages, multiple coders with diverse backgrounds, and sampling until theoretical saturation on which no new concepts were emerging from additional interviews. Second, our study took place during a three-month period in 2015, and we were unable to study whether adoption of the identified factors (use of data, improved community relationships, and enhanced support from zonal or regional health bureaus) would result in improved performance over time, as we had only one year of performance data. Future longitudinal studies of these issues are warranted. Third, we focused on woredas in which performance was in the top 5% and bottom 5% of performance within selected regional and urban/rural sampling frames. Sampling the extremes was a strategy to elucidate and learn from contrasts, particularly to understand the experience of top performing woredas for informing replication and scale up efforts; however, findings may differ in the average performing woredas, which we were unable to examine. Last, we focused on 5 regions of Ethiopia and were unable to examine the experiences of primary care in the pastoral and developing regional states of the country. Although we sought a diverse sample of woredas, our results may have differed if the study were conducted in other regions.

In summary, we found marked differences that distinguished higher-performing and lower-performing woredas in terms of their implementation of primary health care improvements in Ethiopia. These differences pertained to the use of data for problem solving and performance improvement, relationships with the community, and support from higher-level health bureaus that can provide financial, technical, and supervisory support to woreda-level primary health care systems. Focused efforts to strengthen the management and governance of woreda-level primary health care may confer substantial health and health equity benefits as Ethiopia transitions from a lower-income to middle-income country.
